# Birth during on-call period: Impact of care organization on mortality and morbidity of very premature neonates

**DOI:** 10.3389/fped.2022.977422

**Published:** 2022-08-18

**Authors:** Gilles Cambonie, Bénédicte Theret, Maliha Badr, Patricia Fournier, Clémentine Combes, Jean-Charles Picaud, Arthur Gavotto

**Affiliations:** ^1^Department of Neonatal Medicine, Arnaud de Villeneuve Hospital, Montpellier University Hospital Centre, University of Montpellier, Montpellier, France; ^2^Pathogenesis and Control of Chronic Infection, INSERM UMR 1058, University of Montpellier, Montpellier, France

**Keywords:** care organization, morbidity, mortality, on-call period, premature neonate

## Abstract

**Objectives:**

The evidence that risks of morbidity and mortality are higher when very premature newborns are born during the on-call period is inconsistent. This study aimed to assess the impact of this situation among other determinants of outcomes, particularly newborn characteristics and care organization.

**Methods:**

Observational study including all infants born < 30 weeks’ gestation in a French tertiary perinatal center between 2007 and 2020. On-call period corresponded to weekdays between 6:30 p.m. and 8:30 a.m., weekends, and public holidays. The primary endpoint was survival without severe morbidity, including grade 3–4 intraventricular hemorrhage (IVH), cystic periventricular leukomalacia, necrotizing enterocolitis, severe bronchopulmonary dysplasia (BPD), and severe retinopathy of prematurity. The relationship between admission and outcome was assessed by an adjusted odds ratio (aOR) on the propensity of being born during on-call period and expressed vs. weekday. Secondary analyses were carried out in extremely preterm newborns (<27 weeks’ gestation), in cases of early death (within 7 days), and before (2007–2013, 51.5% of the cohort) vs. after (2014–2020, 48.5% of the cohort) the implementation of a pediatrician-nurse team dedicated to newborn care in the delivery room.

**Results:**

A total of 1,064 infants [27.9 (26.3; 28.9) weeks, 947 (760; 1,147) g] were included: 668 during the on-call period (63%) and 396 (37%) on weekdays. For infants born on weekdays, survival without severe morbidity was 54.5% and mortality 19.2%. During on-call, these rates were 57.3% [aOR 1.08 (0.84–1.40)] and 18.4% [aOR 0.93 (0.67–1.29)]. Comparable rates of survival without severe morbidity [aOR 1.42 (0.87–2.34)] or mortality [aOR 0.76 (0.47–1.22)] were observed in extremely preterm infants. The early death rate was 6.4% on weekdays vs. 8.2% during on-call [aOR 1.44 (0.84–2.48)]. Implementation of the dedicated team was associated with decreased rates of mortality [aOR 0.57 (0.38, 0.85)] and grade 3–4 IVH [aOR 0.48 (0.30, 0.75)], and an increased rate of severe BPD [aOR 2.16 (1.37, 3.41)], for infants born during on-call.

**Conclusion:**

In this cohort, most births of very premature neonates occurred during the on-call period. A team dedicated to newborn care in the delivery room may have a favorable effect on the outcome of infants born in this situation.

## Introduction

The quality of patient care depends on several factors, including the availability of caregivers. In many healthcare facilities, however, the caregiving workforce, especially physicians, is reduced at night and on holidays, although the activity may be comparable or even higher than in the daytime and on weekdays. Physical and mental fatigue may, under these conditions, be a pejorative factor in patient care. A study conducted among adult intensivists found that the cognitive abilities of physicians were significantly impaired following a nightshift in the intensive care unit, regardless of professional experience and the length of sleep while on call (1).

In low-risk populations, birth during the on-call period has been associated with adverse perinatal outcomes, notably a lower Apgar score, more frequent admission to a neonatal intensive care unit (NICU), and increased neonatal mortality and morbidity (2–7). This finding may be due to reduced staffing and lower resource availability during on-call periods, particularly for births requiring emergent perinatal interventions. Very premature birth requires a team trained in communication with parents and techniques for the immediate care of these infants. Yet, in this population there is inconsistent evidence of an increased risk of mortality and/or severe morbidity after a birth or NICU admission during off-hours (8–12). These studies, which were based on multicenter cohorts from registers or databases, have variable definitions of the on-call period—frequently limited to the night only—and lack detail on the team managing the patient during off-hour periods. A longitudinal observational study within a single service might better document the impact of the on-call period among the other determinants of patient outcome, particularly the adaptation of care organization and patient characteristics.

The first objective of this study was to assess whether birth during the on-call period was associated with increased mortality or major morbidities in very preterm infants. Our second objective was to determine the impact of the team workforce and composition during these duty-hours on the occurrence of these adverse events.

## Materials and methods

### Population

This observational study was carried out between 1 January 2007 and 31 December 2020, i.e., over 14 years. All live-born premature infants were included if they were born in our institution < 30 weeks’ gestational age (GA), actively managed in the delivery room (DR), and admitted to the department of neonatal medicine (in-born infants).

### Study location

The study was performed in a tertiary perinatal center located at a single site: Arnaud de Villeneuve Hospital, Montpellier University Hospital, France. The median (interquartile range) annual number of births in the center during the period was 3,551 (3,353–3,622), including 14.4 (14.1–14.8)% preterm deliveries.

The DR is located on the ground floor, just below the department of neonatal medicine. Infants are transported in a mobile incubator (Airbone 750i, International Biomedical, Austin, TX) equipped with a ventilator (Fabian, Acutronic Medical Systems, Hirzel, Switzerland), an infusion set (Injectomat Agilia, Fresenius Kabi, Brézins, France), and a cardiorespiratory monitor (IntelliVue X2, Philips Medical Systems, Amsterdam, Netherlands) over an average distance of approximately 150 m. The elevator transport duration, from triggering of the call to the arrival on the first floor is 20–30 s.

The department of neonatal medicine has a capacity of 47 beds, including 14 type 3 beds, corresponding to the NICU, 18 type 2b beds, and 15 type 2a beds. The medical team is composed of nine senior pediatricians, three fellows, and five residents. The number of nurses per neonate is framed by the French perinatal decrees of 1998, i.e., one nurse for two neonates for type 3 beds, one nurse for three neonates for type 2b beds, and one nurse for six neonates for type 2a beds.

### Institutional policy for the care of infants born at 22–29 weeks’ gestation

According to the perinatal protocol of our institution, the care was overwhelmingly palliative for live-born premature infants of 22–23 weeks’ GA, and intensive for infants of 24–29 weeks’ GA.

### Team workforce and composition to provide care in the delivery room

Epoch 1 (January 2007 to December 2013): On weekdays, a pair composed of a senior pediatrician or a fellow and a resident was assigned to provide care for newborns in the DR. During on-call periods, a senior pediatrician or a fellow and a resident were present on site to provide care for infants hospitalized in the department, as well as for those in the DR. Another senior pediatrician could be mobilized for operational duty if necessary.

Epoch 2 (January 2014 to December 2020): A nurse systematically participated in the care of newborns in the DR, on weekdays and during on-call periods. Operational duty was replaced by the on-site presence of a second senior pediatrician. Thus, medical organization was unchanged on weekdays; however, in the second epoch, one of the two pediatricians was fully available, along with the resident and the nurse, to provide care in the DR during on-call periods.

### The main organizational differences between the two epochs were:

– the systematic participation, on weekdays and on-call periods, of a nurse to provide care for newborns in the DR in Epoch 2, whereas this healthcare professional was not involved in Epoch 1;
– the systematic participation, on weekdays and on-call periods, of a pediatrician to provide care for newborns in the DR in Epoch 2, whereas this healthcare professional was sometimes unavailable during on-call periods, and replaced by a resident, in Epoch 1.

During the two epochs, midwives gave routine care to the newborns in the DR. However, when a newborn was considered vulnerable, following a premature birth, for example, they could urgently and even prenatally summon the resident and senior pediatrician by following a systematic appeal procedure. While waiting for their arrival, the midwives could initiate initial care, including warming, drying, stimulation, positioning of the upper airways, providing ventilatory assistance (Neopuff, Fisher and Paykel, Auckland, New Zealand), and even resuscitating the newborn. Thereafter, they participated in the continuation of care under the direction of the pediatric team and ensured the communication of information between the parents and the obstetric team.

### Data collection

Perinatal data for each premature infant were collected prospectively in a database created for neonates born before 33 weeks’ GA and/or with a birthweight less than 1,500 g and admitted to the department of neonatal medicine. The database uses a predetermined form, with detailed definitions of each item. More than 150 variables were assessed for each infant concerning antenatal history and the neonatal period.

### Definitions

The on-call period corresponded to weekdays night, i.e., between 6:30 p.m. and 8:30 a.m., weekends and public holidays. Births during off-peak hours occurred between 12:00 a.m. and 6:59 a.m. (12).

Anthropometric data, including weight, height, and head circumference, were recorded at birth and hospital discharge and expressed as Z scores, according to Olsen et al. (13).

Severe morbidity was defined by at least one of the following conditions (14): (a) severe bronchopulmonary dysplasia (BPD), defined as administration of oxygen for at least 28 days plus the need for 30% or more oxygen and/or invasive mechanical ventilation or continuous positive airway pressure at 36 weeks’ postmenstrual age (15); (b) grade 3–4 intraventricular hemorrhage (IVH) (16) or cystic periventricular leukomalacia (PVL) (17); (c) stage 2–3 necrotizing enterocolitis (NEC) (18); and (d) severe retinopathy of prematurity (ROP), defined as stage 3 or more and/or laser treatment (19).

Severe cerebral lesion was defined by the presence of a grade 3–4 IVH and/or a cystic PVL on cranial ultrasonography.

Early death was defined as occurring within 7 days (5, 7–11). The primary cause of death was classified according to Patel et al. (20).

### Outcomes

The primary endpoint was survival without severe morbidity.

The secondary endpoints included rates of mortality before hospital discharge and any of the major morbidities as defined above.

### Statistical analysis

Comparisons of infants born during vs. outside of an on-call period were made using the Fisher, chi-squared, Student, and Wilcoxon-Mann-Whitney tests, as appropriate.

To limit confounding bias, the association between the birth period and the outcome was adjusted on a propensity score. The propensity score was defined as the infant’s probability of being born during the on-call period based on his/her individual observed covariates and estimated with a logistic regression model. In this model, the birth period was the dependent variable and was studied in relation to the baseline antenatal characteristics that were statistically associated with exposure to the following: multiple birth, antenatal glucocorticoid administration, delivery mode, gestational age, sex, intrauterine growth restriction, premature rupture of membranes, and preeclampsia. The relationship between birth during the on-call period and outcomes was expressed by the odds ratio (OR), weighted by the propensity score (aOR), and the 95% confidence interval (CI).

Three subgroup analyses were carried out: in the group of infants born extremely preterm (< 27 weeks’ GA), in cases of early death (within 7 days), and to compare the outcomes in epochs 1 and 2.

Based on a survival rate without major morbidity of 50% reported in our country for this population (21), and with the assumption that 60% of births occur during the on-call period, 810 subjects were necessary to demonstrate a 10% difference in the rate of survival without severe morbidity, with power of 80% and *p* < 0.05.

Statistical analysis was performed with SAS Version 9 software (SAS Institute, Cary, NC). Values are expressed as numbers (%) or medians (Q25, Q75); *p* < 0.05 was considered statistically significant.

### Ethical considerations

Approval of this study was obtained from the institutional review board of Montpellier University Hospital (IRB-MTP-2021-07-202100891).

## Results

### Characteristics of pregnancy and neonates according to the birth period

During the study period, 1,064 infants < 30 weeks’ GA were admitted to the department: 668 during the on-call period (63%) and 396 (37%) on weekdays. The perinatal characteristics of these patients are summarized in Table 1. Preeclampsia and cesarean delivery were less frequent in mothers who gave birth during on-call. Infants born during on-call had lower exposure to a full course of antenatal corticosteroids, higher exposure to premature rupture of membranes and prenatal antibiotics, higher birthweight, and lower incidence of intrauterine growth restriction.

**TABLE 1:** Perinatal characteristics according to the period of birth.

	On-call (*n* = 668)	Weekday (*n* = 396)	*P*
**Antenatal**			
Multiple pregnancy, *n*%	226 (34)	140 (35)	0.689
PROM, *n*%	232 (35)	111 (28)	0.018
Preeclampsia, *n*%	97 (15)	90 (23)	0.001
Diabetes, *n*%	40 (6.0)	37 (9.3)	0.050
Gestational diabetes, *n*%	36 (5.4)	32 (8.1)	0.092
Glucocorticoids, *n*%	635 (96)	386 (97)	0.293
Full course, *n*%	416 (63)	274 (69)	0.039
Antibiotics, *n*%	380 (58)	184 (47)	<0.001
Cesarean delivery, *n*%	439 (66)	310 (78)	<0.001
**Birth**			
Umbilical arterial blood pH	7.3 (7.2, 7.4)	7.3 (7.2, 7.3)	0.235
Female, *n*%	300 (45)	164 (41)	0.277
Gestational age, weeks	27.7 (26.3, 28.9)	27.9 (26.4, 29.0)	0.094
< 27 weeks, *n*%	241 (36)	427 (64)	121 (31)
27–29 weeks, *n*%	275 (69)	0.071	
Birthweight, g	960 (772, 1,160)	920 (740, 1,105)	0.028
Birthweight Z-score	–0.014 (–0.712, 0.628)	–0.154 (–1.095, 0.406)	<0.001
			0.003
IUGR, *n*%	28 (4.2)	35 (8.9)	
5-min Apgar score	8 (7, 10)	8 (7, 9)	0.911
Score < 7, *n*%	159 (24)	92 (23)	0.881
CRIB-II	8 (7, 9)	8 (7, 9)	0.557

Values are numbers (%) or medians (Q25, Q75).

PROM, premature rupture of membranes; IUGR: intrauterine growth restriction, according to Olsen et al. (13); CRIB-II: clinical risk index for babies (2nd version).

The stay in the department of the infants born during the on-call period was characterized by more frequent use of an umbilical venous catheter for parenteral nutrition and cyclooxygenase inhibitor for patent ductus arteriosus. Their length of stay was comparable to that of infants born on weekdays, although with a slightly higher weight at discharge (Table 2).

**TABLE 2:** Neonatal care according to the period of birth.

	On-call (*n* = 668)	Weekday (*n* = 396)	*P*
**Cardiorespiratory**			
Surfactant, *n*%	545 (82)	314 (79)	0.291
PDA treated with COI, *n*%	277 (41)	136 (34)	0.019
Antihypotensive treatments, *n*%	298 (45)	184 (46)	0.610
Postnatal glucocorticoids, *n*%	223 (33)	118 (30)	0.248
Nitric oxide, *n*%	124 (19)	77 (19)	0.808
Supplemental oxygen, days	8.1 (1.0, 33.0)	12.1 (1.3, 33.0)	0.215
**Ventilatory**			
Invasive ventilation, days	3.0 (0.9, 11.0)	4.0 (0.7, 11.0)	0.774
Non-invasive ventilation, days	35.0 (20.0, 48.0)	35.0 (21.0, 49.0)	0.417
CPAP, days	27.0 (14.0, 38.0)	29.0 (15.0, 39.8)	0.370
HFNC, days	13.0 (7.0, 20.0)	13.0 (8.0, 24.0)	0.174
Overall respiratory support, days	42.0 (26.3, 57.7)	44.6 (28.8, 61.0)	0.230
**Nutrition**			
Umbilical venous catheter, *n*%	506 (76)	257 (65)	<0.001
Umbilical venous catheter, days	3.0 (2.0, 5.0)	4.0 (2.0, 5.0)	0.016
Central venous catheter, days	15.0 (8.0, 25.0)	15.0 (9.0, 27.0)	0.094
Parenteral nutrition, days	14.0 (8.0, 25.0)	15.0 (9.0, 26.0)	0.121
**Discharge**			
Length of stay, days	69.0 (54.0, 85.0)	70.0 (54.0, 85.5)	0.876
Corrected gestational age, weeks	38.0 (36.1, 39.4)	38.1 (36.3, 39.5)	0.603
Weight, g	2,510 (2,100, 2,900)	2,390 (2,000, 2,820)	0.044
Zscore weight	–1.06 (–1.57, –0.54)	–1.21 (–1.76, –0.68)	0.007
Length, cm	45.0 (43.0, 47.0)	45.0 (42.0, 47.0)	0.103
Zscore length	–1.54 (–2.08, –0.84)	–1.57 (–2.30, –0.89)	0.061
Head circumference, cm	33.0 (31.5, 34.0)	33.0 (31.0, 34.0)	0.625
Zscore Head circumference	–0.50 (–1.06, 0.06)	–0.56 (–1.20, –0.06)	0.259

Values are numbers (%) or medians (Q25, Q75).

PDA, patent ductus arteriosus; COI, cyclooxygenase inhibitor; HFOV, high-frequency oscillatory ventilation; CPAP, continuous positive airway pressure; HFNC, high-flow nasal cannula.

### Primary endpoint

**FIGURE 1:**
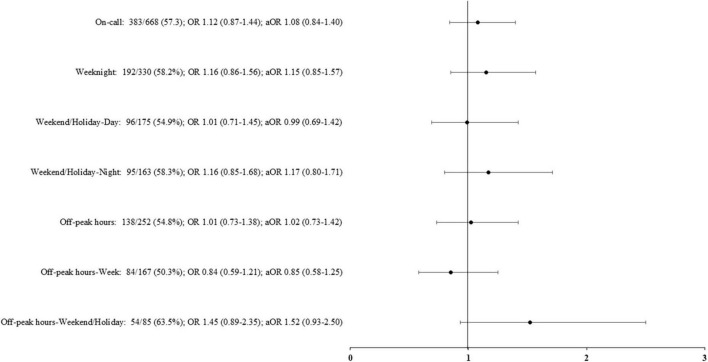
Survival without severe morbidity according to the period of birth. Adjusted odds ratio (aOR), weighted by the propensity score, and their 95% confidence interval shown of the figure, are expressed vs. weekday. On-call: defined as birth in weekdays between 6.30 p.m. and 8.30 a.m., in weekends and public holidays. Off-peak hours: defined as birth between 12:00 a.m. and 6:59 a.m.

For the infants born on weekdays, survival without severe morbidity was 54.5% (216/396). For the infants born during the on-call period, this rate was 57.3% (383/668), aOR (95% CI) 1.08 (0.84–1.40). No difference in either the unadjusted or adjusted analysis of the propensity score was observed for survival without severe morbidity for births in the different on-call subperiods. In addition, birth during off-peak hours, whether on weeknights or holidays, had no significant influence on the primary endpoint (Figure 1).

### Secondary endpoints

**FIGURE 2:**
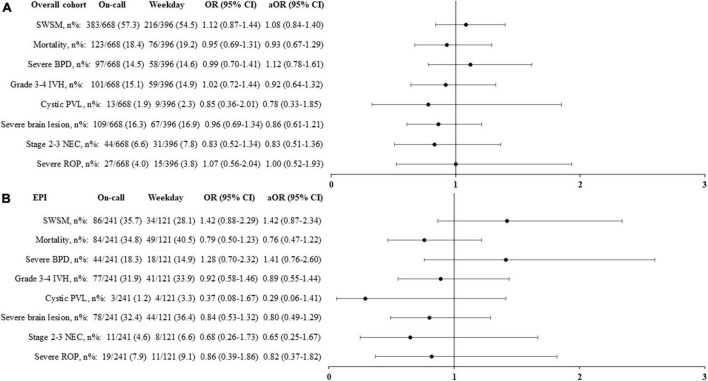
Primary and secondary outcomes according to the period of birth in the overall population **(A)** and in extremely premature infants, born < 27 weeks’ gestational age **(B)**. Adjusted odds ratio (aOR), weighted by the propensity score, and their 95% confidence interval (95% CI) shown of the figure, are expressed vs. weekday. On-call: defined as birth in weekdays between 6.30 p.m. and 8.30 a.m., in weekends and public holidays. EPI, extremely premature infants; SWSM, survival without severe morbidity; BPD, bronchopulmonary dysplasia; IVH, intraventricular hemorrhage; PVL, periventricular leukomalacia; NEC, necrotizing enterocolitis; ROP, retinopathy of prematurity.

No difference in mortality or any major morbidity was observed according to the period of birth in either the unadjusted or the adjusted analysis (Figure 2A).

### Subgroup analysis

#### Infants born extremely preterm

In this cohort, 362 (34%) infants were born < 27 weeks’ GA, including 19 < 24 weeks’ GA. Their median GA and birthweight were, respectively, 25.6 (24.9, 26.3) weeks and 752 (660, 870) g. Survival without severe morbidity, mortality, and major morbidities were unaffected by birth occurring during the on-call period (Figure 2B).

#### Early mortality

**FIGURE 3:**
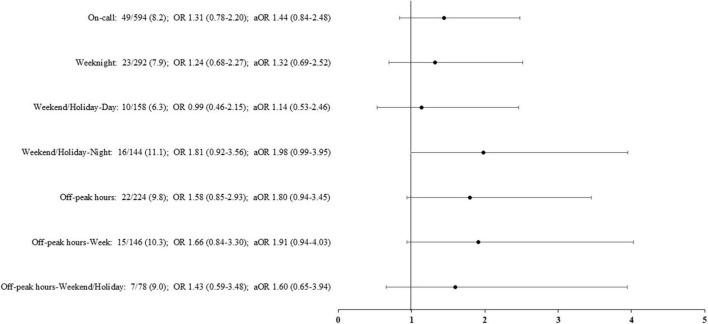
Rates of early death (within 7 days) according to the period of birth. Adjusted odds ratio (aOR), weighted by the propensity score, and their 95% confidence interval shown of the figure, are expressed vs. weekday. On-call: defined as birth in weekdays between 6.30 p.m. and 8.30 a.m., in weekends and public holidays. Off-peak hours: defined as birth between 12:00 a.m. and 6:59 a.m.

Death occurred within 7 days in 71 (6.7%) infants, corresponding to 36% (71/199) of overall mortality. In infants born on weekdays, the early death rate was 6.4% (22/342). The occurrence of this event was unaffected by the birth period (Figure 3), including for birth occurring during the night on weekends or holidays [aOR (95% CI) 1.98 (0.99, 3.91)] and birth during off-peak hours [aOR (95% CI) 1.80 (0.94, 3.45)].

#### Comparison of the two epochs

Perinatal characteristics according to the two epochs are shown in Table 3. Preeclampsia, diabetes, vaginal delivery and birth during the on-call period were more frequent in the second epoch. Infants born during the second epoch had lower exposure to a full course of antenatal corticosteroids and were more frequently female. CRIB-II scores were comparable among groups.

**TABLE 3:** Perinatal characteristics according to the epoch of birth.

	Epoch 1 (*n* = 548)	Epoch 2 (*n* = 516)	*P*
**Antenatal**			
Multiple pregnancy, *n*%	200 (36)	166 (32)	0.196
PROM, *n*%	179 (33)	164 (32)	0.894
Preeclampsia, *n*%	84 (15)	103 (20)	0.043
Diabetes, *n*%	28 (5.1)	49 (9.5)	0.006
Glucocorticoids, *n*%	531 (97)	490 (96)	0.613
Full course, *n*%	374 (68)	316 (62)	0.038
Antibiotics, *n*%	311 (57)	253 (51)	0.054
Cesarean delivery, *n*%	421 (77)	328 (64)	<0.001
**Birth**			
Female, *n*%	220 (40)	244 (47)	0.022
Gestational age, weeks	27.86 (26.43, 28.86)	27.86 (26.14, 28.86)	0.857
< 27 weeks, *n*%	175 (32)	187 (36)	0.154
27–29 weeks, *n*%	373 (68)	329 (64)	
Birthweight, g	950 (760, 1,140)	945 (750, 1,150)	0.712
Birthweight Z-score	–0.05 (–0.89, 0.52)	–0.08 (–0.77, 0.55)	0.919
IUGR, *n*%	31 (5.7)	32 (6.2)	0.700
5-min Apgar score	8 (7, 9)	8 (7, 10)	0.522
Score < 7, *n*%	124 (23)	127 (25)	0.470
CRIB-II	8 (7, 9)	8 (7, 10)	0.262
On-call, *n*%	326 (59)	342 (66)	0.026
< 27 weeks, *n*%	114 (65)	127 (68)	0.654

Values are numbers (%) or medians (Q25, Q75).

PROM, premature rupture of membranes; IUGR: intrauterine growth restriction, according to Olsen et al. (13); CRIB-II: clinical risk index for babies (2nd version).

**FIGURE 4:**
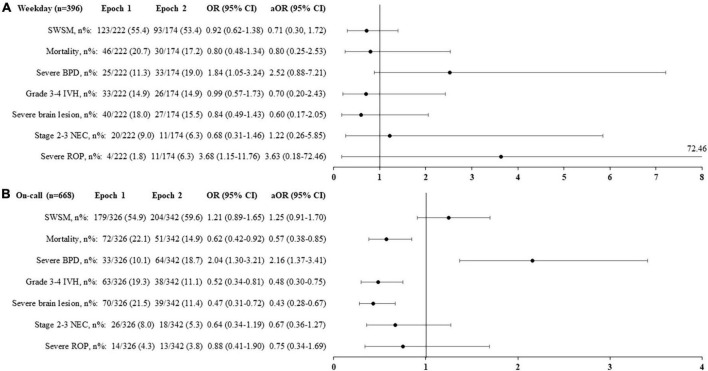
Primary and secondary outcomes according to the epochs and the period of birth [Weekday: **(A)**, On-call: **(B)**] in the overall population. Adjusted odds ratio (aOR), weighted by the propensity score, and their 95% confidence interval (95% CI) shown of the figure are expressed vs. Epoch 1. On-call: defined as birth in weekdays between 6.30 p.m. and 8.30 a.m., in weekends and public holidays. SWSM, survival without severe morbidity; BPD, bronchopulmonary dysplasia; IVH, intraventricular hemorrhage; NEC, necrotizing enterocolitis; ROP, retinopathy of prematurity.

For infants born on weekdays, the adjusted analyses revealed no significant change between the two epochs in rates of survival without severe morbidity, mortality, or severe morbidities (Figure 4A).

For infants born during on-call, epoch 2 was associated with decreased rates of mortality [aOR (95% CI) 0.57 (0.38, 0.85), *p* = 0.03], grade 3–4 IVH [aOR (95% CI) 0.48 (0.30, 0.75), *p* = 0.03], and severe brain lesions [aOR (95% CI) 0.43 (0.28, 0.67), *p* < 0.001], as well as an increased rate of severe BPD [aOR (95% CI) 2.16 (1.37, 3.41), *p* = 0.03] (Figure 4B).

**FIGURE 5:**
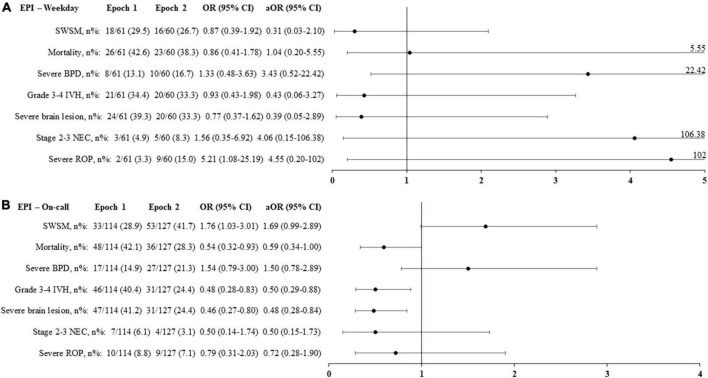
Primary and secondary outcomes according to the epochs and the period of birth [Weekday: **(A)**, On-call: **(B)**] in extremely premature infants (born < 27 weeks’ gestational age). Adjusted odds ratio (aOR), weighted by the propensity score, and their 95% confidence interval (95% CI) shown of the figure are expressed vs. Epoch 1. On-call: defined as birth in weekdays between 6.30 p.m. and 8.30 a.m., in weekends and public holidays. EPI, extremely premature infants; SWSM, survival without severe morbidity; BPD, bronchopulmonary dysplasia; IVH, intraventricular hemorrhage; NEC, necrotizing enterocolitis; ROP, retinopathy of prematurity.

For extremely preterm infants born during on-call, epoch 2 was associated with decreased rates of grade 3–4 IVH [aOR (95% CI) 0.50 (0.29, 0.88), *p* = 0.009] and severe brain lesions [aOR (95% CI) 0.48 (0.28, 0.84), *p* = 0.005]. The mortality rate did not change significantly [aOR (95% CI) 0.59 (0.34–1.00), *p* = 0.06] (Figures 5A,B).

Causes of death also differed; notably, mortality associated with severe cerebral lesions was reduced in the overall population in the second epoch (Table 4).

**TABLE 4:** Causes of death according to the epochs in the overall population, and in extremely premature infants (born < 27 weeks’ gestational age).

	Epoch 1	Epoch 2	*P*
Overall population	(*n* = 118)	(*n* = 81)	0.02
Respiratory failure, *n*%	19 (16)	16 (20)	
Severe brain lesions, *n*%	59 (50)	21 (26)	
NEC, *n*%	10 (8)	7 (9)	
Infection, *n*%	14 (12)	15 (19)	
Immaturity, *n*%	9 (8)	11 (14)	
Congenital anomalies, *n*%	7 (6)	8 (10)	
Others, *n*%	0 (0)	3 (4)	
**Extremely premature infants**	**(*n* = 74)**	**(*n* = 59)**	**0.12**
Respiratory failure, *n*%	12 (16)	14 (24)	
Severe brain lesions, *n*%	41 (55)	19 (32)	
NEC, *n*%	3 (4)	4 (7)	
Infection, *n*%,	10 (14)	8 (14)	
Immaturity, *n*%	6 (8)	10 (17)	
Congenital anomalies, *n*%	2 (3)	2 (3)	
Others, *n*%	0 (0)	2 (3)	

Values are numbers (%).

NEC, necrotizing enterocolitis.

## Discussion

This study showed that most births of very preterm infants occurred during the on-call period. While these births differed in several perinatal characteristics from those occurring on weekdays, no impact was observed on survival or the occurrence of severe morbidities during hospitalization, including for extremely premature birth. Our study also suggested that organizational factors, such as the availability and composition of the team providing care in the DR, influenced the risk of death and some severe morbidities in cases of very premature birth.

We defined the on-call period to include nights, weekends and public holidays because there are fewer physicians at these times and the team is less experienced compared with the traditional pair of a senior physician and a resident. In a conventional day/night segmentation (day: 8:30 am–6:30 pm; night: 6:31 pm–8:29 am), it is important to note that 70% of the time in a week corresponds to the on-call period (118 out of 168 h), and this ratio is even higher in weeks with public holidays. Therefore, it was unsurprising that most of the admissions in our sample occurred during this period. Few studies have adopted a comparable definition and have found birth occurrence or NICU admission rates ranging from 60 to 67% (5, 10).

The ratio of births during the on-call period could not be explained only by the relative length of this period over a week. As observed (10), infants born on weekdays were more likely to be born after a scheduled cesarean birth and a full course of glucocorticoids in the context of preeclampsia or intrauterine growth retardation. Conversely, births during on-call were less planned, as suggested by the higher rates of vaginal delivery and exposure to antenatal antibiotics. In our study, this resulted in newborns with a modestly lower GA for births occurring during on-call and a lower birthweight Z-score for births occurring on weekdays. These differences in perinatal characteristics had a negligible impact on the care provided in the NICU and no impact on survival without severe morbidity, mortality or the components of severe morbidity, despite the multiplicity of approaches of the on-call period.

Studies carried out in NICU networks in Canada and Australia have found contradictory results on the risk of early mortality among infants < 32 weeks’ gestation admitted at night. These differences may be related to organizational differences within units and within networks. Indeed, a network with the prerogative to regulate perinatal traffic may avoid excessive workloads in individual units, which can be a source of excessive mortality (9, 10, 22). Our night admission rate, 45%, did not suggest an excessive workload compared to the rates close to 60% reported by institutions whose organization entailed a slightly longer night period of 15 h (9, 11). About 23% of our population was admitted during off-peak hours, between 12:00 p.m. and 6:59 p.m., a rate consistent with those reported in the United States (12, 23). The trend found in this study toward a higher risk of early death for births occurring during off-peak hours is consistent with observations from California and Pennsylvania, which suggests that structural factors, such as lower staffing levels and greater nighttime fatigue, expose patients to increased risks of mortality or severe morbidity during this period (12).

A recent report from the Canadian Neonatal and Preterm Birth networks suggested a higher mortality in the event of evening birth (4:00 p.m. to 11:59 p.m.) in extremely premature infants of 22–27 weeks’ gestation (24). This finding was interpreted as possibly resulting from obstetrical features, notably a higher rate of acute pregnancy complications including chorioamnionitis, placental abruption, premature rupture of membranes and precipitous labor. In our study, no association was found between birth during on-call and crude or adjusted odds of mortality or major morbidities among infants < 27 weeks’ gestation. It should be noted, however, that our data included very few infants born < 24 weeks. The general policy in our country is to propose palliative care but not intensive care in this situation (25).

Early mortality, before 7 days, comprised only 34% of the mortality observed in our sample during hospitalization. This rate was identical to that observed in the French EPIPAGE-2 study, a prospective population-based cohort of very preterm births performed in 2011 (26). This timeline prompted us to consider death occurring during the entire hospital stay, because organ failure and the adequacy of care provided in the few hours following birth may influence survival beyond the first postnatal week. The rate, timing, and causes of death observed in this study seem consistent with the data observed in our country (25), suggesting a more pessimistic view of the prognosis for very preterm infants, with a lower threshold for transitioning to palliative care, particularly in cases of severe brain lesions (27).

The composition and availability of the team caring for the patients during epoch 2 seemed clearly more appropriate, with a physician-nurse pair dedicated to this activity. The presence of a nurse trained in the department of neonatology ensured expertise for carrying out technical procedures in the DR, such as the placement of peripheral venous catheters; the initiation of intravenous fluids, caffeine and—if required—antibiotics or sedatives; the preparation and assistance for surfactant administration; and the set-up of non-invasive and invasive ventilation devices (28). Monitoring the infant’s temperature, oxygenation, and glycemia was also more rigorous during this second period. Depending on the training courses for healthcare professionals in different countries, and the organization of departments in hospital centers, all or part of these missions can be carried out by other providers, notably respiratory therapists. Previous studies have suggested the importance of the quality of care provided during the stabilization phase, referred to as the “Golden Hour,” for the mortality and morbidity of very preterm infants (29–32).

Several factors may explain why improvements in death and severe brain lesion rates were observed only for on-call periods. Numerically, change in the organization of care in the DR was mainly applied to neonates born during the on-call period, since their rate increased significantly during the second epoch. In addition, this new organization also had several consequences for the dispensation of care by the pediatrician. First, the physician was more fully available, with the possibility of concentrating full attention on the infant in the DR, since a second pediatrician was present on site for the other patients hospitalized in the department. Second, the composition of the pair was more homogeneous, since it was always a senior pediatrician who provided care and no longer a resident, who could be called upon during the first period in the event of the senior’s unavailability. Third, the second senior on site could, if available, assist in the newborn’s care. In our experience, this process was sometimes easier to mobilize during on-call than on weekdays. Fourth, the presence of two seniors on site brought serenity and security and reduced the physicians’ stress. It also allowed each one to take a few minutes or hours of rest by alternating these rest times, which can restore mental capacities and performance (33).

Our data found an increased rate of severe BPD in infants born on-call during epoch 2. Other prospective longitudinal registries have reported increased incidence of BPD, whereas in-hospital mortality had decreased over time among very preterm infants (34–36). Potential explanations include the increased survival of the most immature infants, who are the most at risk of severe BPD, changes in respiratory care (37, 38), and a more restrictive use of postnatal corticosteroids (39).

### Limitations

Our study has several limitations. The results of this retrospective, single-center study, which only considered patients born in a tertiary perinatal center and managed in a single NICU, may be difficult to extrapolate to other centers. Compared to population-based studies, however, it offers other advantages. Our data collection was homogeneous since it was carried out by only two investigators. The number of missing data items was very low, enabling us to present factual results with no imputed or projected data. Above all, we had in-depth knowledge of organizational factors, notably the resources and variations in staffing patterns, for both physicians and nurses, according to on-call or weekday periods, as well as the different epochs of the observational period.

Maternal, obstetric and neonatal data were linked, which is not the case for all databases (24). We analyzed the main maternal characteristics, however we did not evaluate all obstetric care and we cannot exclude that some of them varied over time. The prolonged study period, 14 years, was also associated with various changes in protocols and daily clinical practice in neonatology. Thus, the suggested relationships between care organization and patient outcomes are hypothetical. The main limitation of observational studies is residual confounding, and this requires to interpret the results with great caution.

Another weakness is our limited number of patients. However, for the hypothesis tested, we greatly exceeded the number of subjects required. In addition, the result for the primary endpoint was very close to the rate observed in the national EPIPAGE-2 cohort, for infants of comparable gestational ages (21).

It is also likely that active care was not provided for most of the births occurring at 22–23 weeks. Mortality in the DR despite intensive management of these newborns was not reliably documented over the study period. For this reason, we did not adjust our sample based on birth certificates from our institution, as some livebirths may have been misclassified and recorded as stillbirths since intensive care was not provided (25). We opted to focus our data on infants admitted to our NICU, which more clearly manifests a deliberate intention to initiate active treatment.

## Conclusion

This study showed that the births of the most vulnerable newborns occurred mainly during the on-call period, i.e., at moments when medical resources were suboptimal, both quantitatively and qualitatively. This finding suggests that each NICU should individually analyze the features of the population admitted during this period and adjust to ensure adequate organization. Our experience suggests that a physician-nurse pair specifically dedicated to the initial care in the DR may ease the situation in units with high patient volume and staff workload and prolonged workshifts during on-call periods. This might improve the prognosis for patients and enhance job satisfaction for caregivers and reduce the risk of burnout.

## Data availability statement

The original contributions presented in this study are included in the article/supplementary material, further inquiries can be directed to the corresponding author/s.

## Ethics statement

The studies involving human participants were reviewed and approved by the Institutional Review Board of Montpellier University Hospital (IRB-MTP-2021-07-202100891). Written informed consent from the participants’ legal guardian/next of kin was not required to participate in this study in accordance with the national legislation and the institutional requirements.

## Author contributions

GC, BT, MB, PF, J-CP, and AG conceptualized and designed the study, contributed to the search for published works, carried out the data acquisition and interpretation, and drafted and finalized the report. CC performed the data analysis, contributed to the data interpretation, and critically revised the report. All authors have read and approved the final manuscript.

## Abbreviations

aOR: adjusted odds ratio
BPD: bronchopulmonary dysplasia
CI: confidence interval
DR: delivery room
GA: gestational age
IVH: intraventricular hemorrhage
NEC: necrotizing enterocolitis
NICU: neonatal intensive care unit
PVL: periventricular leukomalacia
ROP: retinopathy of prematurity.
